# Comparison of exercise and physical activity routine and health status among apparently healthy Nigerian adults before and during COVID-19 lockdown: a self-report by social media users

**DOI:** 10.1186/s42269-022-00815-y

**Published:** 2022-05-27

**Authors:** Patrick Ayi Ewah, Adetoyeje Y Oyeyemi, Adewale L Oyeyemi, Saturday N Oghumu, Peter Agba Awhen, Mary Ogaga, Lucy Inyang Edet

**Affiliations:** 1 Department of Physiotherapy, Faculty of Allied Medical Sciences, University of Calabar, Calabar, Nigeria; 2 Department of Medical Rehabilitation (Physiotherapy), Faculty of Allied Health Sciences, University of Maiduguri, Maiduguri, Nigeria

**Keywords:** Exercise participation, Physical activity, Perceived health, Covid-19 pandemic, Lockdown

## Abstract

**Background:**

COVID-19 is a pandemic that ravaged the world in manners that were never seen in the recent past, and one of the measures to stem the tide of this ravaging pandemic is a stay-at-home order referred to as lockdown. This study compares the physical activity status and perceived health of Nigerians before and during the lockdown.

**Results:**

Social media platform users (*n* = 205) were surveyed using a two-part questionnaire. The first part elicited the sociodemographic characteristics of the subject. In the second part, information about their exercise and physical activity, general health, and economic palliatives as offered by the government, non-governmental organizations, and philanthropists were elicited. The frequency of exercise was significantly more (*p* < 0.05) during the lockdown than before the lockdown. The duration and intensity of the exercise per week were comparable. There was also a negative relationship between the body mass index, frequency, and duration of exercise before and during the lockdown. The subjects perceived their health as worse during (3.70 ± 1.05) the lockdown than before (3.95 ± 0.97) lockdown (*Z* = − 3.69, *p* = 0.00).

**Conclusion:**

Overall, for these cohorts of social media platform users, lockdown did not adversely affect their exercise routine. It is recommended that there should be specific recommendation on exercise as an important Instrumental Activity of Daily Living [IADL]. Therefore, while this pandemic lockdown lasts and beyond, the safety measures to follow while partaking in this IADL should be included in the public health recommendation.

## Background

On the 28th of February, 2020, Nigeria announced the first confirmed case of COVID-19, the novel SARS-Cov-2 coronavirus (Lai et al. 2020), and became the first West African country with a confirmed case of the novel COVID-19 (Olurounbi and Bala-Gbogbo 2020). This was followed by the World Health Organization (WHO) declaration that the novel coronavirus outbreak is a public health emergency of international concern, and that the daily estimates were expected to rise (Health Emergency Office 2020; Johns Hopkins University 2020; WHO 2020). Following the outbreak of the coronavirus pandemic, governments of different countries began to take swift and protective measures to contain the spread, (Chen et al. 2020) and in Nigeria, the National Center for Disease Control led the initiatives to manage the pandemic through measures including isolation, quarantine, testing, treatment, monitoring, and surveillance (Nigerian Centre for Disease Control 2020).

In Nigeria, the administrative and commercial capital cities of Lagos and Abuja and the state of Ogun in southwest Nigeria were placed on an initial 2-week lockdown. Subsequently, the details of COVID-19 measures were publicized, and the lockdown became extended to other cities and towns in the states. The lockdown measures included, but are not exclusive to travel warnings/bans, flight cancellations, stay-at-home orders, closing down of both public/private schools, closing down of houses of worships, and suggestions that workers on non-essential duties should work from home**.** Other details, such as social distancing, frequent washing of hands or the use of alcohol-based sanitizers, wearing of face mask, avoidance of touching the eyes, nose, and mouth, were announced.

Health authorities advised people to stay at home (Nigerian Centre for Disease Control 2020)**,** although an average working Nigerian leaves home early in the morning daily in the week, and most of them walk for long or short distances, to work or to the farm, or to take transport to work. It is a general belief that staying at home can promote sitting or lying down for an extended period watching television, excessive use of mobile phones with possible deleterious effect on the health of such individuals. For example, an increase in sitting time and sedentariness, may worsen the health condition of those with a chronic disease condition, and may reduce the quality of life (Chen et al. 2020; Owen et al. 2010) of even a healthy adult when the lockdown is relaxed.

Regular participation in physical activity and exercise (walking, jogging, cycling, and dancing) has many health benefits (Cha et al. 2016). Active living prevents diseases such as hypertension, arteriosclerosis, and diabetes (Fletcher et al. 1996; National Institution of Health 1998). More so, active living may also have other potential positive effects such as improved pulmonary function, and cardiovascular, and aerobic fitness, stronger immunity, enhanced muscle strength, higher bone density, stress reduction, and better emotional stability (Cha et al. 2016). We have also been able to show elsewhere that the rate of recovery of the heart rate and blood pressure following a bout of exercise is more in active individuals compared to the inactive individuals (Oyeyemi et al. 2015). It is recommended that adults should undertake at least 30 min of moderate physical activity every day or at least 15 min of vigorous physical activity every day for at least 5 days a week (U.S. Department of Health and Human Service 2018).

Recent reports suggest that there is increasing awareness of the health-enhancing benefits of physical activity and exercise among Nigerians (Umeifekwen 2011). Anecdotal reports show that in many Nigerian cities in the morning, it is common to observe some young adults engaging in walking or jogging. It is the general belief that weekend mornings are especially the most common time people engage in exercise. Lack of exercise and hypo-activity can exacerbate any preexisting mental health condition, such as anxiety and depression, and may lead to substance abuse, domestic violence, sexual violations, and crimes (Cao et al. 2020; Zhang et al. 2020). Other than individual initiatives and expert advice to media audiences, no advice or recommendation on physical activity behavior during the pandemic and especially during lockdown was issued by the National Center for Disease Control or any government agency in Nigeria.

Lockdown is a life-saving and survival measure that is a necessity but it is a general belief that it impacts nations' economies negatively (Lima et al. 2020). The measure that restricts citizens to their homes also brings untold hardship to the general population. Many Nigerians were not able to earn any income, and palliatives are hardly enough succor where such is offered. Worldwide there is some consensus on the economic consequences of any lockdown. However, the health consequences of lockdown are not given much attention or are out rightly ignored. Lockdown restricts movement and invariably also restricts outdoor exercise, activities in public parks, and fitness gyms. However, how the restrictions of movement during the lockdown due to COVID-19 affect Nigerian people is unclear, as there is hardly any empirical data on this especially in Nigeria.

The social media platforms such as Facebook, linked-in, WhatsApp and Messenger are today's forums of interaction by people of similar interests or dispositions. Social media platform users constitute a sizeable population of youths and adults who are often highly educated and are homogenous groups constituting a significant sub-population of any nation in the contemporary world (Ngonso 2019). Through this platform, information is disseminated speedily among group members. Albeit inappropriately, this platform has become the means through which even official workplace communication is disseminated to employees, and health information including exercise/physical activity instruction, and advice are shared through support groups via the social media platform (Leonardi et al. 2013; Baruah 2012).

A physically active lifestyle is habituated and requires self-discipline to keep, and adhere to, and is presumed to be uncultivated by many people. For example, a lifestyle of physical activity is presumed to be low across the population especially in urban areas worldwide (Guthold et al. 2018). Information on how the lockdown impacts the physical activity status of the populace is important to assess the possible overall effect of the lockdown on any population. How the lockdown measures in Nigeria impact the physical activity pattern or behavior of Nigerians is unclear. The objective of this study was therefore to compare the pattern and level of physical activity behavior of Nigerians before and during the period of lockdown. It was hypothesized that there will be no significant difference between the frequency of exercise before and during COVID-19 Lockdown, there will be no significant difference between the duration of exercise before and during COVID-19 Lockdown, and there will be no significant relationship between the health status of the subject before and during COVID-19 Lockdown.

## Methods

### Subjects

The subjects for this study were 18–60 years old. The recruited subjects were Nigerians, who can read and write in English, and frequently make use of any of the social media (Facebook, WhatsApp, Messenger, or Linked-in). The subjects were also residents in Nigeria when the data were collected. Nigerians who were sick or under any form of treatment or medications for an ailment or who were hospitalized or on lockdown outside the country were excluded from participation.

### Instrumentation

The main instrument for this study is a researcher-developed Physical Activity Survey on COVID-19 Lockdown. The first part of the 16-item survey sought sociodemographic information including age, occupation, the current state of residence, self-reported height, and weight. In the second part, subjects were asked about their participation in physical activity/exercise, the type, duration, and frequency of exercise, and were also asked to rate themselves on their health status before and during the COVID-19 lockdown. They were also asked about their level of satisfaction, and happiness or, unhappiness during the lockdown. Finally, they were asked whether they received any palliative measures from any government, non-government organization, or any individual philanthropist.

The instrument was reviewed by a university lecturer with not less than 10 years of experience in teaching and research in physiotherapy and community practice that includes instrument design and adaptation. Five other professionals in the discipline of physiotherapy with not less than 10 years of practice and teaching experience attested to the face validity of the instrument. The reliability of the instrument was examined by checking internal consistency using the Cronbach alpha coefficient. Test–retest reliability and correlation analysis were also conducted for examining the reliability of the instrument. The internal consistency reflects the homogeneity of the questionnaire, with a Cronbach α of 0.60. The instrument was tested for reliability by having 16 adults in the age range of 18–56 complete the questionnaire using Spearman correlation (Twee et al. 2007). The questionnaire was completed twice with a duration of one week between the first and second administration. There was a high degree of correlation between first and second administration (*r* = 0.89, *p* < 0.05). The questionnaire was tested using reliability analysis and the spearman test at *α* = 0.05.

### Procedure

Ethical approval was sought from the Research and Ethical Committee of the University of Maiduguri, College of Medical Sciences, before the commencement of the study. In this online survey, the Physical Activity Survey on COVID-19 questionnaire was shared through social media platforms including Facebook, WhatsApp, Messenger, and Linked-in platforms. Subjects who consent to participate were advised to click on a link that was sent and were advised to read the preliminary instructions before deciding to participate in the survey.

In the preliminary instruction, subjects were invited to participate. Invited subjects were informed that participation is voluntary and that no name was required on the survey. In addition to assuring them of anonymity, they were also informed that any data obtained will be pooled together with those of others and will be treated in aggregates only and that no harm will be sustained during participation.

In addition to previous information, prospective subjects were informed that if they consent to participate, the survey should be completed and that they can decline participation by not completing the survey without any punishment or penalty. No gifts or reward was pledged for the subjects, but the subjects were informed that the results of the study will be shared with anyone who indicated interest, when appropriate, and only upon request. Upon return of the survey questionnaire, all completed and usable responses were entered into a computer for statistical analysis. Parametric and nonparametric data were treated as appropriate for descriptive and inferential analysis of the data.

## Data analysis

Data obtained were entered into a computer and were analyzed using the Statistical Package for Social Scientists (SPSS, version 20). Descriptive statistics of mean, standard deviation, and frequencies were used to summarize the sociodemographic and physical characteristics of the data. The data were subjected to a test for normality using Kolmogorov–Smirnov test, and normally distributed variables were analyzed with paired/independent t test (for differences) and Person’s correlation (relationship test), and non-normal data were analyzed using spearman's correlation, Wilcoxon signed-rank, and Chi-square (test for a relationship, difference, and association). These analyses were used to explore trends of relationship, associations, and the difference between the frequency, intensity, and time duration of exercise, as well as other applicable data on participation in physical activity and the perceived health status of subjects before and during COVID-19 lockdown. The level of significance was set at *p* < 0.05.

## Results

### Physical characteristics of the subjects

A total of 205 subjects who were physiotherapists, medical laboratory scientists, Civil servants, students, housewives, and businessmen participated in the study. A simple majority of the subjects were male (53.7%, *n* = 110), and 46.3% (*n* = 95) were female. The mean age, weight, and height of the subjects were 31.79 ± 8.38 years, 70.52 ± 15.33 kg, and 1.68 ± 0.12 m, respectively. A simple majority (49.3%) of the subjects were civil servants (*n* = 101). The mean duration in minutes, it took the subjects to complete the electronic survey was 12.18 ± 18.66 min (Table 1). Seventy-one (34.6%) of the subject engaged in walking exercise, and 30.2% engaged in jogging (*n* = 62). Some subjects about 22.9% (*n* = 47) engaged in a combination of aerobic, weight lifting, and abdominal exercises, or play sports like badminton. Only a few of the subject engaged in dancing (*n* = 16), skipping (*n* = 5), cycling (*n* = 3), and swimming (*n* = 1**). **A majority of the subjects were of ages 18–30 (*n* = 97), followed by 31–40 (*n* = 80), 41–50 (*n* = 24) and 51–60 (*n* = 4).

**Table 1:** Physical Characteristics of the Subjects (values are in mean and SD)

Variables	Mean/SD	CV
Age (years)	31.79 ± 8.38	0.26
Weight in kilogram (kg)	70.52 ± 15.33	0.22
Height meter (m)	1.68 ± 0.12	0.07
Body Mass Index (kg/m^2^)	25.18 ± 5.76	0.23
Frequency of exercise before lockdown (days/week)	3.04 ± 1.78	0.59
Freq. exercise during the lockdown (days/week)	3.32 ± 2.01	0.61
Duration exercise before the lockdown (minutes/day)	37.27 ± 33.89	0.91
Duration exercise during the lockdown (min/day)	33.59 ± 29.20	0.87
Intensity of exercise before the lockdown (min/week)	135.52 ± 183.63	1.36
Intensity of exercise during the lockdown (min/week)	128.55 ± 137.35	1.07
Completion time (min)	12.18 ± 18.66	1.53

CV, coefficient of variation (standard deviation divided by mean)

A majority of the subject either strongly disagree (*n* = 83) or disagree (*n* = 70) that they are happy that they were in a lockdown, and 3.9% (*n* = 8) either strongly agree or agree (21.5%; *n* = 44). A simple majority of the subject also strongly disagree (*n* = 102) or disagree (*n* = 62) that they are happy with the palliative measures provided by the government while others agree (*n* = 35) or strongly agree (*n* = 6) that they are happy. Also, a majority of the subject strongly disagree or disagreed (*n* = 183) that they had received any of these palliative measures provided during the lockdown while only a few either agreed or strongly agreed (*n* = 22).

### Comparison, relationship, and association before and during COVID-19 Lockdown

The number of days during which the subjects exercise or engage in physical activity was significantly higher (*p* = 0.03) during the lockdown than before the lockdown (3.32 ± 2.01 vs. 3.04 ± 1.78 days), but there was no difference in duration of exercise (*p* = 0.10) before (37.27 ± 33.89) and during (33.59 ± 29.20) the COVID19 lockdown. There was, however, a significant strong positive relationship between the duration of exercise before and during the lockdown (*r* = 0.50; *p* = 0.00). A significant strong relationship was also found between the frequency of exercise before and during the lockdown (*r* = 0.53; *p* = 0.00). On the contrary, a significant medium positive relationship was found between the intensity of exercise before and during the lockdown (*r* = 0.39; 0.00). There was also a negative relationship (not significant) between the BMI and duration of exercise before and during the lockdown (Table 2). Hence, an increase in BMI results in a decrease in the duration, frequency, and intensity of exercise albeit not to a significant level. The perceive health of subjects was significantly better (*Z* = − 3.69, *p* = 0.00) before (HBLD = 3.95 ± 0.97) the lockdown than during (HDLD = 3.70 ± 1.05) the lockdown. There was also a small negative (not significant) relationship between being Happy with Lockdown—health Before Lockdown, Happy with Palliative Measure—Health During Lockdown, and Receive Palliative Measure/health before and during the lockdown; however, there was a small negative but significant relationship (*r* = − 0.15; *p* = 0.03*) between being Happy with Palliative Measure-Health Before Lockdown, and relationships between other variables can be seen in Table 3. There was also a highly significant association between HappLD—HappPM (*p* = 0.00), HappLD—RecPM (*p* = 0.00), HappPM—RecPM (*p* = 0.00), and HBLD—HDLD (*p* = 0.00) (Table 4). However, there was no significant association (*p* > 0.05) between HappLD—HBLD; HappLD—HDLD; HappPM—HBLD/HDLD (Table 4).

**Table 2:** Relationship between Body Mass Index, duration of exercise before and during lockdown, frequency of exercise before and during, and intensity of exercise before and during COVID-19 lockdown

Variables	*R* values	*P* values
BMI (kg/m^2^)-DEXBLD	− 0.01	0.86^NS^
BMI (kg/m^2^)-DEXDLD	− 0.09	0.21^NS^
BMI (kg/m^2^)-FEXBLD	− 0.04	0.60^NS^
BMI (kg/m^2^)-FEXDLD	− 0.10	0.14^NS^
BMI (kg/m^2^)-INTEXBLD	− 0.02	0.74^NS^
BMI (kg/m^2^)-INTEXDLD	− 0.07	0.35^NS^

DEXB/DLD, duration of exercise before and during lockdown; FEXB/DLD, frequency of exercise before and during lockdown; INTEXBLD, intensity of exercise before and during lockdown; BMI (kg/m^2^), Body Mass Index in kilogram; NS, not significant

**Table 3:** Relationship between happy with lockdown, happy with palliative measure, receive palliative measure, health before and during COVID-19 lockdown

Variables	*r* values	*P* values
Happy with lockdown-happiness palliative measure	0.32	0.00**
Happy with lockdown-receive palliative measure	0.22	0.00**
Happy with lockdown-health before lockdown	− 0.09	0.20^NS^
Happy with lockdown- health during lockdown	0.18	0.01*
Happy with palliative measure-receive palliative measure	0.54	0.00**
Happy with palliative measure- health before lockdown	− 0.15	0.03*
Happy with palliative measure- health during lockdown	− 0.04	0.62^NS^
Receive palliative measure- health before lockdown	− 0.14	0.05^NS^
Receive palliative measure- health during lockdown	− 0.05	0.51^NS^
Health before lockdown- health during lockdown	0.51	0.00**

**Highly significant *p* < 0.01 level (2-tailed)

*Significant at *p* < 0.05 level (2-tailed)

NS, not significant *p* > 0.05

**Table 4:** Association between happy with lockdown, happy with palliative measure, receive palliative measure, health before and during COVID-19 lockdown

Variables	Values	*df*	Phi	Cramer’s V	*p* values
HappLD – HappPM	60.13	9	0.54	0.31	0.00*
HappLD – RecPM	24.10	9	0.34	0.20	0.00*
HappLD-HBLD	15.95	12	0.28	0.16	0.19^NS^
HappLD- HDLD	14.74	12	0.27	0.16	0.26^NS^
HappPM-RecPM	145.00	9	0.84	0.49	0.00*
HappPM – HBLD	16.91	12	0.29	0.17	0.15^NS^
HappPM- HDLD	19.47	12	0.31	0.18	0.08^NS^
RecPM- HBLD	16.41	12	0.29	0.16	0.17^NS^
RecPM-HDLD	10.16	12	0.22	0.13	0.60^NS^
HBLD- HDLD	170.00	16	0.91	0.46	0.00*

HappLD, happy with lock down; HappPM, happy palliative measure; RecPM, receive palliative measure; HBLD/HDLD, health before and during lockdown

*p < 0.05; NS = *p* > 0.05

### Gender differences: duration, frequency, intensity, height, weight, body mass index before and during the lockdown

The difference in the duration, frequency, intensity, height, weight, and body mass index of the subjects before and during the lockdown is presented in Table 5. The duration of exercise before (42.04 ± 34.60 min/day vs 31.76 ± 32.35 min/day) and during (38.21 ± 30.71 min/days vs 28.24 ± 26.5 min/day) the lock down was significantly higher (*p* < 0.05) in the male subjects than the female subjects. There was no significant difference (*p* > 0.05) in the frequency of exercise before the lockdown although during the lockdown the frequency of exercise in the male subjects was significantly more (*p* < 0.05) than that of the female subjects. Other variables such as intensity of exercise, weight, height and BMI are also presented in Table 5.

**Table 5:** Gender differences: duration, frequency, intensity of exercise before and during lockdown

Variables	Male	Female	CV	*p* values
Duration of exercise BLD (min/day)	42.04 ± 34.60	31.76 ± 32.35	1.02	0.03*
Duration of exercise DLD (min/day)	38.21 ± 30.71	28.24 ± 26.51	0.94	0.01*
Frequency exercise BLD (days/week)	3.24 ± 1.67	2.81 ± 1.88	0.6	0.09^NS^
Frequency of exercise DLD (days/week)	3.60 ± 1.84	2.99 ± 2.14	0.72	0.03*
Intensity of exercise BLD (min/weeks)	157.63 ± 188.79	109.92 ± 179.99	1.64	0.06^NS^
Intensity of exercise DLD (min/weeks)	148.82 ± 143.79	105.07 ± 126.22	1.20	0.02*
Height (meter)	1.72 ± 0.11	1.63 ± 0.11	0.07	0.00*
Weight (kg)	72.18 ± 13.62	68.59 ± 16.95	0.25	0.09^NS^
BMI (kg/m^2^)	24.60 ± 5.27	25.84 ± 6.25	0.24	0.13^NS^

BLD, before the lockdown; DLD, during the lockdown; CV, coefficient of variation

*p < 0.05 significant; NS, not significant, value are mean ± standard deviation

## Discussion

Lockdown is a potent measure of saving a life that was employed throughout the world, and many countries (China, Korea, Italy, USA) in the forefront of the pandemic adopt this measure, one way or the other either wholly or partially (Moodie 2020; Zhang et al. 2020). Lockdown, although a life-saving measure with success in containing the spread of COVID-19. The measure impacts negatively on the nation’s economy and in turn the individuals, as it creates untold hardship and dwindles the ability to earn income (Lima et al. 2020) which may be implicated for the poor overall health as reported in the present study. This study set out a tone on how a stay-at-home order by the government impacts the lifestyle of its citizenry.

Majority of the subjects in this study were young adult between the age of 18–30 years old. This finding was, consistent with that of Ngnoso, which attest to the fact that the social media platform users constitute a size-able population of the young adult (Ngonso 2019). The finding in the present study is also consistent with that of Jan Hruska and Maresova who found out that the above age group made use of social media more frequently, and that as age increases the number of users of social media decreases (Hruska and Maresova 2020).

The subjects in this study performed exercise more frequently during the lockdown than before the lockdown but there was no difference in the duration, and intensity of the exercise. On average, lockdown duration only modestly but insignificantly exceeded the recommendation on physical activity (U.S. Department of Health and Human Services 2018). The increased frequency of exercise during as against before the COVID-19 Lockdown as seen in the present study may be attributed to the use of coping strategies by the subjects to avoid the deleterious effect of lockdown. A strong positive relationship between the duration and frequency of exercise before and during the COVID-19 lockdown signifies that an increase in the frequency of exercise before COVID-19 lockdown also increases duration and frequency of exercise during the lockdown. A negative relationship (non-significant) between BMI, duration, frequency, and intensity of exercise before and during COVID-19 lockdown as reported in the present study signifies that as BMI increases the duration, frequency, and intensity of exercise also reduces concurrently albeit not to a significant level. It has been shown that an increase in the frequency and duration of physical activity resulted in greater weight loss, fat loss, and reductions in measures of central obesity (Slentz et al. 2004).

Hence, we inferred that subject with higher BMI tends to spend less duration, intensity, and frequency during exercise before and during the lockdown.

Lockdown especially during a global pandemic like COVID-19 turns a once energetic individual into becoming hypoactive and reduced the quality of life of even the healthiest individual hence worsening chronic condition; (Cha et al. 2016; Nigerian Centre for Disease Control 2020) such individuals may be left with no option than increasing the number of days in a week they engage in exercise. Our hypothesis that there will be no difference in the frequency of exercise before and during the pandemic lockdown is therefore rejected.

Increasing the frequency of exercise during the lockdown may be regarded as a coping mechanism. However, unfortunately, this coping mechanism did not prevent the feeling of poor health during the lockdown perhaps due to the modesty of the improvement in the frequency which is not enough to meet the health-enhancing exercise, and physical activity recommendation of at least 5 days a week, and 30 min per session (U.S. Department of Health and Human Services 2018). It could also be that the magnitude of the impact of the lockdown on health is too much more than a modest improvement in exercise participation could counteract. The finding in the present study is consistent with a previous study by Grasdalsmoen et al. which concluded that a large majority of young adults fail to meet international recommendations on exercise (Grasdalsmoen et al. 2019).

The modest but insignificant improvement in the duration of participation in physical activity is good but it still could not have resulted in improved well-being or health by the subjects. This finding is therefore not in accord with the consensus that increased participation in physical activity should lead to an improvement in perceived well-being and overall health status (psychological well-being) of subjects (Butt et al. 2016). Our hypothesis that there would be no significant difference in the duration of exercise before and during the lockdown is therefore not rejected.

It is also notable that the overall health perception is worse during the pandemic lockdown than it was before the lockdown. On a good note, however, this study suggests that there may not be any recidivism in the exercise habit cultivated before the lockdown and during the pandemic lockdown among this cohort of social media platforms users. A significantly poorer health status during the lockdown than before the lockdown as observed in the present study is consistent with finding by Lima et al. who reported that measure which disrupts people's jobs and lives could take a heavy toll on people's health and well-being (Lima et al. 2020). Also, our hypothesis that there will be no significant difference in the health status before and during the lockdown is therefore rejected.

Exercise and health-enhancing physical activity are important IADL that are recommended for all adults to stem the tide of chronic lifestyle diseases such as diabetes, obesity, and heart diseases, which is now regarded as a pandemic even worldwide. It is unknown whether the subjects in this study exercise in groups, alone, indoor in the gym or they exercise outdoor. Nonetheless, it is important to include social distancing and other measures such as enforced compliance with gym occupancy rate when exercising indoor, avoiding overcrowding indoor, and outdoor as well as observe other measures to prevent the spread of any COVID-19 and infectious diseases in the context of the current pandemic.

### Study limitations and Strength

The result of this study should be interpreted in place of some limitations. First, the use of cross-sectional data, which precludes establishing a temporal and causal comparison between physical activity and the rating of perceived health among this cohort of social media users. Second, the self-reported value on height, weight, and health may not reflect true measure as subjects may not know their actual values on the physical characteristics. We also could not call the subjects to confirm whether provided values were correct. Lastly, exercise frequency and durations are estimates and may be more or less than the values reported. Nevertheless, this study provides some insights into the experience of a cohort of social media platform users that may be fairly generalizable to a not insignificant sub-population of Nigerians, during the COVID -19 pandemic.

### Conclusions

The present study showed that the number of days in which the subjects engage in physical activity was significantly higher during the COVID-19 lockdown than before the lockdown, but no difference was observed between the duration of exercise before and during the lockdown. The subjects in the present study also showed a significant poorer health during the COVID-19 lockdown than before the lockdown. However, there was a significant high association between I have received palliative during the lockdown and being happy during the lockdown.

## Data Availability

Data and material are available upon request.

## Abbreviations

COVID: Coronavirus disease
BMI: Body Mass Index
IADL: Instrumental activity of daily living
WHO: World Health Organization
DEXB/DLD: Duration of exercise before and during lockdown
FEXB/DLD: Frequency of exercise before and during lockdown
INTEXBLD: Intensity of exercise before and during lockdown
HappLD: Happy with lock down
HappPM: Happy palliative measure
RecPM: Receive palliative measure
HBLD/HDLD: Health before and during lockdown
USA: United State of America
